# Moth wings as sound absorber metasurface

**DOI:** 10.1098/rspa.2022.0046

**Published:** 2022-06

**Authors:** Thomas R. Neil, Zhiyuan Shen, Daniel Robert, Bruce W. Drinkwater, Marc W. Holderied

**Affiliations:** ^1^ School of Biological Sciences, University of Bristol, Bristol, UK; ^2^ Department of Mechanical Engineering, University of Bristol, Bristol, UK

**Keywords:** acoustic metamaterial, biological sound absorber, deep-subwavelength, bioinspired metamaterials

## Abstract

In noise control applications, a perfect metasurface absorber would have the desirable traits of not only mitigating unwanted sound, but also being much thinner than the wavelengths of interest. Such deep-subwavelength performance is difficult to achieve technologically, yet moth wings, as natural metamaterials, offer functionality as efficient sound absorbers through the action of the numerous resonant scales that decorate their wing membrane. Here, we quantify the potential for moth wings to act as a sound-absorbing metasurface coating for acoustically reflective substrates. Moth wings were found to be efficient sound absorbers, reducing reflection from an acoustically hard surface by up to 87% at the lowest frequency tested (20 kHz), despite a thickness to wavelength ratio of up to 1/50. Remarkably, after the removal of the scales from the dorsal surface the wing's orientation on the surface changed its absorptive performance: absorption remains high when the bald wing membrane faces the sound but breaks down almost completely in the reverse orientation. Numerical simulations confirm the strong influence of the air gap below the wing membrane but only when it is adorned with scales. The finding that moth wings act as deep-subwavelength sound-absorbing metasurfaces opens the door to bioinspired, high-performance sound mitigation solutions.

## 1. Introduction

The performance of an acoustic absorber depends on how its thickness relates to the longest wavelengths for which it is designed. Traditionally, acoustic absorbers have implemented porous and fibrous materials to achieve absorption [1], or perforated panels implementing tuned cavity depths [2]. These designs suffer from either imperfect impedance matching to the incoming wave, or the necessity to be large, with dimensions comparable to the target wavelength. At lower frequencies, these sound absorbers must therefore become increasingly bulky, with porous sound absorbers being effective at thicknesses above *λ*/10 [3]. Acoustic metamaterials have been designed and constructed that offer substantial efficiency gains through the realization of deeply subwavelength acoustic absorption [4–6]. In practice, acoustic metamaterial absorbers typically consist of a periodic grid of tuned resonators with a total thickness much thinner than that of the working wavelength [7,8]. Recent technical advances in deep-subwavelength acoustic absorbers have resulted in absorbers consisting of structures with a feature size as low as *λ*/223 [9]. While these metamaterials provide strong low-frequency sound absorption at impressive thickness to wavelength ratios, they are narrowband, covering only tens of hertz around their operating frequency [4]. Other low-frequency (50–400 Hz) metamaterials have developed that function over wider bandwidths (approx. one octave band) yet these metamaterials have larger thickness to wavelength ratios of around *λ*/8 to *λ*/45 [10–12].

Metamaterials were originally thought not to occur naturally, yet in several instances, the process of adaptive evolution has harnessed the desirable phenomena achievable by metamaterials. A remarkably high proportion of these have been found in the order Lepidoptera (butterflies and moths): the scales on some butterfly wings, such as those of the morpho blue, contain photonic crystals that, for example, create striking blue structural coloration [13,14], and some moth silk can reflect and guide broadband wavelengths of light [15]. Furthermore, there has been a growing interest in bioinspired metamaterials, with researchers looking to nature for clues into designing the next generation of advanced metamaterials. This has been particularly prevalent in the world of dissipative metamaterials. Designs have been realized that mimic the hierarchical structure of natural materials such as shells [16,17] and the periodic structure of spider silk [18] that facilitate tuneable elastic wave attenuation.

Lepidopteran scales are identified here to offer a productive evolutionary playground for natural metamaterials as moth scales have also been recognized as forming a naturally occurring acoustic metamaterial able to absorb sound at a very low thickness-to-wavelength ratio (approx. *λ*/100) [19–21]. The wings of moths are decorated with scales of varying size, each with its own resonant frequency [19,21]. Each scale absorbs sound at the frequency of its main resonance modes [21]. When numerous scales of differing size and therefore resonant frequencies cover the membrane, the result is broadband acoustic absorption in the deep-subwavelength regime [19].

Previous work on moth scales has characterized the absorption brought about by the scales when the wing membrane and associated scales were backed by air [19]. This is the common predator–prey scenario where echolocating bats depend on echo reflections from flying prey. In this situation, a sound absorber coating reduces the prey's detectability by bat biosonar. Here, we explore the sound-absorbing capabilities of moth wings when they are placed upon an acoustically reflective substrate. Thereby we explore their effectiveness as a sound absorber metasurface, ultimately aiming at architectural application of biomimetic sound absorbers [4]. To do this, we ensonified a solid metallic disc with or without a coating by an (intact or modified) moth wing segment to measure its spectral and directional effect on sound reflections.

Specifically, we predicted that an intact layer of moth wing reduces the spectral reflection coefficient (RC) significantly, showing deep-subwavelength performance. We further hypothesized that the two layers' respective absorptions both contribute to the overall absorption performance. The importance of direct sound exposure to the scales was tested by characterizing wing samples with one scale layer removed with the scales either facing the sound or the reflective surface.

We then developed semi-empirical numerical models employing the finite element method (FEM) to replicate the functionality, hence developing a deeper theoretical understanding of the mechanisms at work. The ultimate aim of understanding the evolved acoustic absorption mechanisms is to explore their potential for bioinspired acoustic metamaterials in the frequency range of the human ear.

## 2. Material and methods

### (a) Moths

**Figure 1.**
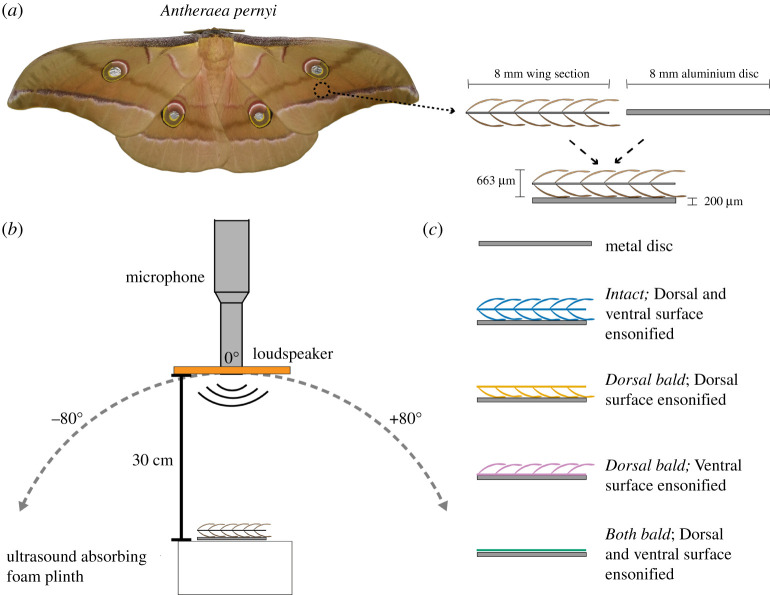
Location of wing punch taken from the moth species *Antheraea pernyi* (*a*). Experimental set-up for characterizing the angular distribution of RC of the wing sample and metal disc (*b*). Workflow of the six experimental treatments (*c*). (Online version in colour.)

Pupae of the moth species *Antheraea pernyi* (Guerin-Meneville, 1855) were obtained from wwb.co.uk from May–June 2019. Pupae were housed in a temperature-controlled cabinet (Economic Deluxe, Snijders Scientific, Tilburg, Holland), where they were subject to a 12-hour night/day cycle in which temperature varied between 25°C and 30°C while humidity was a constant 70%. Following eclosion, specimens were euthanized by freezing them at −18°C. One circular section was punched from the centre of one forewing (figure 1*a*) of each specimen with an 8 mm diameter biopsy punch (Kai medical, Japan). These circular wing samples were used for further analysis.

### (b) Morphometrics of the scale layer

Scanning electron microscopy (SEM) images (Zeiss Evo15 with Lab6 emitter, Zeiss, Germany) were used to characterize individual scales and their arrangement on the wing sample. Wing samples were mounted on adhesive carbon tabs (EM Resolutions Ltd, UK) and coated with 5 nm of gold (Quorum Q150R ES, Quorum Technologies Ltd, UK). Samples were imaged in both high-vacuum mode using an SE1 detector and variable pressure mode using a VPSE G3 detector. An applied electron high tension of 15–20 kV with 50–100 pA probe and a magnification range from × 250 to × 5 k were used. The morphology of individual base scales and cover scales (*n *= 5 of both scale types for each of the five wing samples) was characterized by six parameters (see electronic supplementary material, table S1): ‘scale length’ measured from base to tip of the scale; ‘scale width’ as greatest width measured perpendicular to the long axis and ‘aspect ratio’ as length divided by width. Scale microstructure comprises parallel longitudinal ridges connected by cross-ribs, which were characterized by ‘inter-ridge distance’ and ‘cross-rib distance’. Finally, ‘layer thickness’ for ventral and dorsal surfaces and base as well as cover scales was measured from wing sections imaged from the side as a distance from the wing membrane to the tip of the furthest scale (see electronic supplementary material, table S1). All image analyses were performed using ImageJ (ImageJ, NIH, USA).

### (c) Reflection coefficient measurements

Sound reflection measurements followed [19]. Measurements were taken in a 2.9 × 2.7 × 2.3 m semi-anechoic single wall audiometric room (IAC Acoustics, North Aurora, Illinois). The measurement head consisted of a 1/4" (6.4 mm) ultrasound microphone with the protective grid removed (type 26AB, GRAS Sound & Vibration A/S, Holte, Denmark), a pre-amplifier (type 2669L), a power supply (type 5935-L, both Brüel & Kjær, Nærum, Denmark) and a custom-made ring-shaped electret foil loudspeaker (Emfit Ltd., Vaajakoski, Finland; outer radius 12 mm, hole radius 7 mm) driven by a PZD350 M/S high-voltage amplifier (TREK Inc., Lockport, NY). The microphone was positioned in the central circular opening of the ring speaker with speaker and microphone membrane in the same plane pointing at the acoustic centre of the set-up from a distance of 30 cm (figure 1*a*). The acoustic axes of microphone and speaker were thus coaxial.

The target object was placed in the centre of the set-up on a plinth (75 × 70 × 25 mm) made of ultrasound-absorbing foam (Basotect W, BASF, Ludwigshafen, Germany) that is non-reflective across the entire frequency range tested here. A cross-line laser level (FatMax 77–153, Stanley, UK) was used to align the centre of the object with the acoustic centre of the set-up to ensure consistency between specimen placements (figure 1*b*).

Objects were ensonified with linear frequency modulated sweeps from 250 to 15 kHz of 10 ms duration. Sweeps were sampled using a microphone at 500 kHz with 16-bit resolution. Playback and recordings were sample-synchronous at the same sampling rate and resolution. Recorded sweep echoes were converted into impulse responses using pulse forming through the complex spectral division with the echo recorded perpendicularly from a 50 × 70 cm metal plate (calibration target).

Echo measurements were taken from a circular aluminium disc of 8 mm diameter either on its own or covered by a circular wing sample of the same size and shape (*n *= 5). Each wing sample on the disc was ensonified first with the dorsal surface facing upwards towards the incident sound and second with the ventral surface facing upwards (*Intact* treatments). Then, all scales on the dorsal side of the wing sample were removed using a section of ultrasound-absorbing foam fashioned into a pointy tool (*Dorsal bald*) and the same dorsal and ventral measurements were taken. Finally, all scales on the ventral surface were removed leaving only the wing membrane (*Both bald*) and again dorsal and ventral measurements were taken. This resulted in six different measurements: (i) *Intact*, dorsal surface ensonified; (ii) *Intact*, ventral surface ensonified; (iii) *Dorsal bald*, dorsal surface ensonified; (iv) *Dorsal bald*, ventral surface ensonified; (v) *Both bald*, dorsal surface ensonified and (vi) *Both bald*, ventral surface ensonified (figure 1*c*). When a bald surface was to be in contact with the metal disc (i.e. measurements iv, v and vi), a drop of water was used to seal the membrane to the metal disc to ensure there was no air trapped underneath the membrane.

To measure the reflection directionality of the object, the measurement head was mounted on a computer-controlled LT360 turntable (LinearX Systems Inc., Battle Ground, WA) allowing ensonification and echo measurement of the object placed at the acoustic centre of the set-up from a range of incidence angles (figure 1*b*). We measured echoes from 80° either side (−80° to +80°) of the direction of normal sound incidence (0°) in 0.5° steps. We then turned the impulse responses taken from all these directions into a tomographic image of the sample by an inverse Radon transform. To remove background noise, we then manually selected the image area containing the target object and then applied a Radon transform to extract only the aspects of the echo impulse responses originating from the target (for details see [22]). All spectral analyses, including for normal sound incidence, were based on echo impulse responses processed this way.

Microphone, loudspeaker and turntable were connected to and controlled by a NI-DAQ BNC2110 card operated through LabVIEW v. 16.0 (both National Instruments) using custom-written scripts. All digital sound processing was performed using MATLAB (v9, MathWorks, Natick, MA).

### (d) Calculation of spectral target strength and reflection coefficient

Target strength *TS* was calculated as follows:

$$TS=\text{10lo}{\text{g}}_{\text{10}}\left(\frac{I_{r}}{I_{i}}\right)\text{, }$$

where *I_i_* (W m^−2^) is the incident sound intensity reaching the target and *I_r_* the returned sound intensity at 0.1 m distance. We corrected for the measurement distance of 30 cm by adding 3.54 dB for spherical spreading losses.

Measured echo impulse responses were selected manually and zero-padded to a length of 2048. Spectral target strength was then calculated by fast Fourier transform (FFT, 2048 point rectangular window).

The RC of the wing samples was then calculated using

$$RC=\frac{I_{r}}{I_{i}}.$$

### (e) FEM modelling

We created two FEM models to match our two sets of empirical data: spectral effects for normal sound incidence and the directionality of target strength. Three-dimensional FEM models of simplified scales on a wing membrane were built in COMSOL Multiphysics (v5.3a, COMSOL Inc., Burlington, MA) aiming to quantitatively recreate the measured spectral RCs.

**Figure 2.**
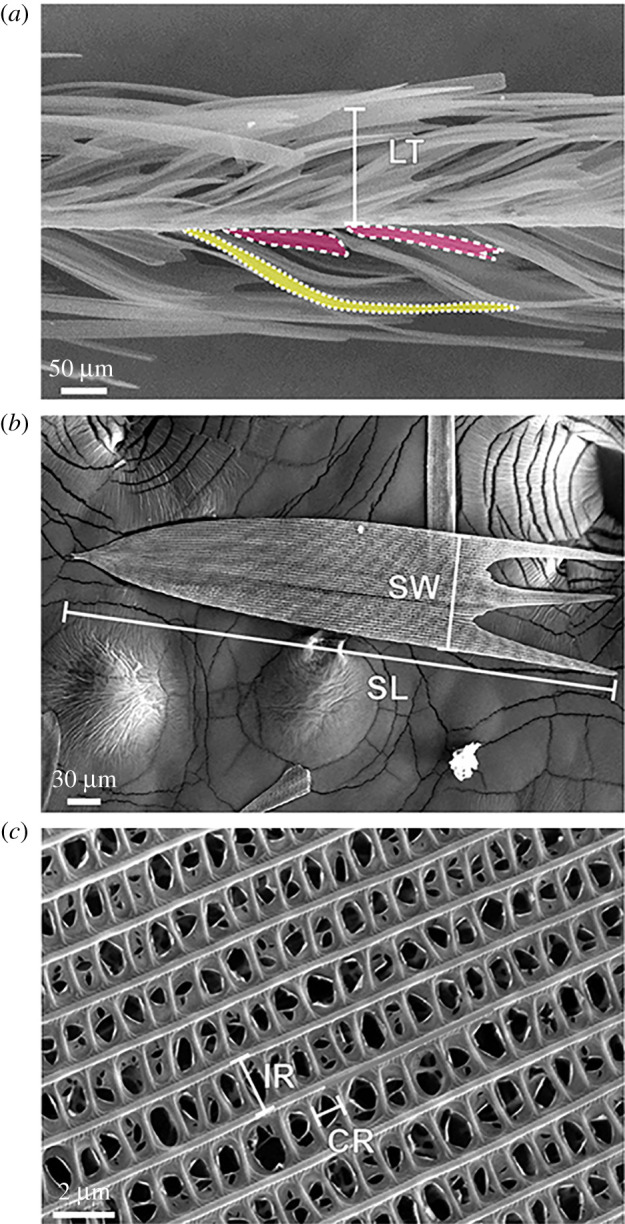
Scanning electron microscopy image of (*a*) Cross-section through the target wing section highlighting two base scales (pink, white dashed outline) and one cover scale (yellow, white dotted outline). (*b*) Single base scale in top view. (*c*) Microstructure of a base scale showing the parallel ridges and cross-ribs. Layer thickness (LT), scale length (SL), scale width (SW), inter-ridge distance (IR) and cross-rib distance (CR). (Online version in colour.)

To model normal sound incidence, the model unit cell contained a single scale representing either a dorsal base scale (base scales form the layer closest to membrane; pink in figure 2*a*) or a dorsal cover scale (cover scales overlap base scales and form top layers; yellow in figure 2*a*). Periodic boundary conditions were implemented on the side walls to expand the unit cell into an infinite two-dimensional array. Background acoustic plane waves were impinged from a direction normal to the array to mimic echo recordings from normal sound incidence. RC was calculated by dividing the scattered wave intensity sent back into the direction of sound incidence (backscatter) by the input wave intensity. Four treatments were calculated that replicate four of the measurement treatments: (i) *Intact*, dorsal surface ensonified; (iii) *Dorsal bald*, dorsal surface ensonified; (iv) *Dorsal bald*, ventral surface ensonified and (v,vi) *Both bald*.

A second scale array model was built to calculate the reflection directionality of the scale array (electronic supplementary material, figure S1). The model contains a single 8 mm long row of scales with average dimensions enclosed in a disc-shaped air domain. Periodic boundaries were used to expand the model into an infinite two-dimensional array. The model hence was not an 8 mm disc but rather an 8 mm strip. The incident wave was again a background plane wave incident from −90° to +90° with 0° representing normal sound incidence. The scattered field returned in the incident wave direction (backscatter) was used to calculate the reflection calculation. Reflection directionality was calculated from −90° to +90° above the array in 1° steps which is a slightly wider range of incidence angles and half the angular resolution used for directionality measurements. For effective material properties and full details, see [19,21].

### (f) Statistical analyses

All statistical analysis was performed using a commercial statistical analysis package (R studio v. 0.99.473, RStudio, Inc. Boston, MA) and statistical significance was accepted as *p* < 0.05. A Shapiro–Wilk normality test was used to assess whether the data followed a normal distribution. Repeated measures *t*-tests (two-tail) were used to compare differences in target strengths and RCs among treatments as a function of frequency. The data are displayed as means with standard error, *n* refers to number of individuals used per species, as indicated in each legend.

## 3. Results

### (a) Scale layer morphometrics

Both surfaces of the wing had an understorey of shorter base scales covered by more elongated cover scales (figure 2*a*). Specific scale morphologies are summarized in electronic supplementary material, table S1. The total layer thickness was 663.79 ± 51.24 µm, corresponding, at 20 kHz, to a ratio of absorber thickness to sound wavelength of 1/26 for an entire intact wing and 1/50 when the dorsal scales were removed.

### (b) Spectral target strength and reflection coefficient for normal sound incidence

#### (i) Dorsal ensonification

**Figure 3.**
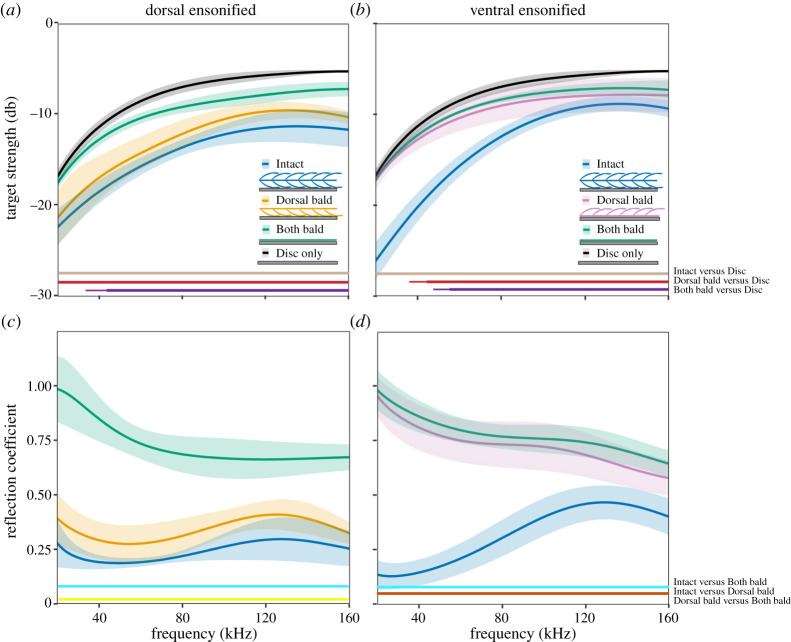
Spectral target strength (*a*,*b*) and RC ((*c*,*d*); mean and standard error as shaded area; *n* = 5) of a metal disc covered by a wing sample for three experimental treatments (Intact, Dorsal bald and Both bald) when ensonifying either the dorsal surface (*a*,c) or the ventral surface (*b*,*d*). Horizontal lines near abscissa indicate significant pairwise differences (thin lines *p *≤ 0.05; thick lines *p *≤ 0.01). (Online version in colour.)

First, we measured spectral sound reflections with the dorsal surface of the wing sample facing the incident sound at normal sound incidence. The presence of the wing sample on the metal disc reduced the target strength depending on treatment and as a function of frequency: (i) *Intact:* spectral target strength ranged from −11.3 dB to −22.2 dB which is significantly lower than the disc alone (−5.2 dB to −16.6 dB; target strength difference = 5.7 dB–7.2 dB) across all frequencies (20–160 kHz). (iii) *Dorsal bald*: there was a similar reduction in spectral target strength relative to the disc −9.5 dB to −21.2 dB (target strength difference = 4 dB–5.7 dB). Finally, (v) *Both bald*: there were no significant differences in target strength at lower frequencies (less than 35 kHz) but target strength was 0.7 dB to 2.3 dB lower than for the disc alone at higher frequencies (figure 3*a*).

The corresponding spectral RC of the metal disc covered by the *Intact* wing sample was 0.27 at 20 kHz (total layer thickness/*λ* = 1/26) and ranged between 0.18 and 0.3. The *Intact* wing sample on the disc reduced the RC significantly more than the *Both bald* wing sample over the entire frequency range measured (0.67–0.98), while removing only one layer of scales *Dorsal bald* resulted in a non-significant increase in RC (0.27–0.41, dorsal layer thickness/*λ* = 1/50) compared to the *Intact* sample across the entire frequency spectrum (figure 3*c*).

#### (ii) Ventral ensonification

When the wing samples were ensonified with their ventral surface facing the incident sound, spectral target strength of the *Intact* sample was between −8.8 dB and −26 dB which is significantly lower than the disc alone (−5.2 dB to −16.6 dB; target strength difference = 3.4 dB–9.6 dB) across all frequencies tested (20–160 kHz). The *Dorsal bald* had significantly higher target strengths (−7.8 dB to −17 dB) compared to the *Intact* treatment over all frequencies tested but had significantly lower target strengths than the disc alone at frequencies greater than 37 kHz (0.4 dB–2.7 dB). Finally, the *Both bald* treatment showed no significant differences in target strength at lower frequencies (less than 42 kHz), but was 0.5 dB to 2.1 dB lower in target strength at higher frequencies than the disc alone (figure 3*b*).

The corresponding spectral RC with the *Intact* wing sample covering the metal disc was again significantly lower than the *Dorsal bald* and *Both bald* treatments over the entire frequency range measured (0.13 at 20 kHz, total layer thickness/*λ* = 1/26). There were no significant differences between the *Dorsal bald* and the *Both bald* treatments when the sample was ensonified ventrally (figure 3*d*).

#### (iii) Modelling the effect of base and cover scales on reflection coefficient

**Figure 4.**
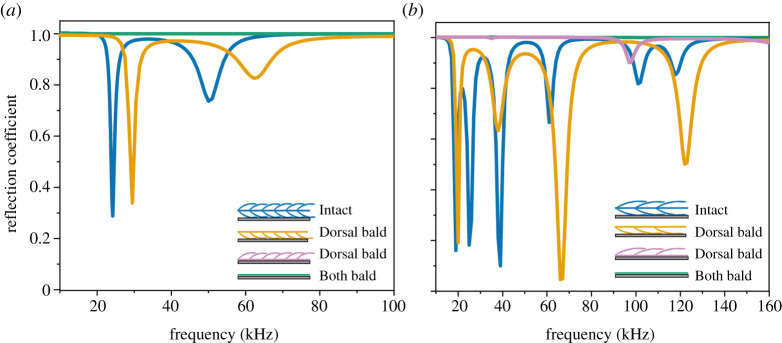
Calculated spectral RCs of a wing segment decorated with (*a*) base scales and (*b*) cover scales of average dimensions (see electronic supplementary material, table S1) in four treatments: ‘Intact’; ‘Dorsal bald, dorsal surface ensonified’; ‘Dorsal bald, ventral surface ensonified’; ‘Both bald’ (see schematics in legends; note: Both bald curve covers Dorsal bald, ventral surface ensonified curve in (*a*)). (Online version in colour.)

Scale length and width used for modelling were based on the average value of the cover and base scale measured from the SEM images (electronic supplementary material, table S1). Numerical simulations of dorsally ensonified *Intact* and *Dorsal bald* base scales show two RC sinks (low reflection), which correspond to the frequencies of two resonance modes of the base scales (figure 4*a*). Remarkably, the same *Dorsal bald* sample ensonified ventrally shows 100% reflection at all frequencies as does the *Both bald* sample (figure 4*a*). Similar patterns in the same four treatments were found for an array of cover scales (figure 4*b*). The dorsally ensonified *Dorsal bald* and *Intact* array both show a series of RC sinks, while both the ventrally ensonified *Bald* and *Dorsal bald* treatments show full reflection at all frequencies (RC = 1). In comparison, there were more RC sinks for cover than for base scales, and they changed more widely between treatments. Note that the *Intact* layer of cover scales has two RC sinks both around 20 kHz and around 110 kHz. These are the result to somewhat different average cover scale morphologies in the dorsal and ventral scale layers (electronic supplementary material, table S1). No double sinks are present for base scales because average dorsal and ventral base scales are morphologically similar and resonate at very similar frequencies.

#### (iv) Modelling the effect of an air gap

**Figure 5.**
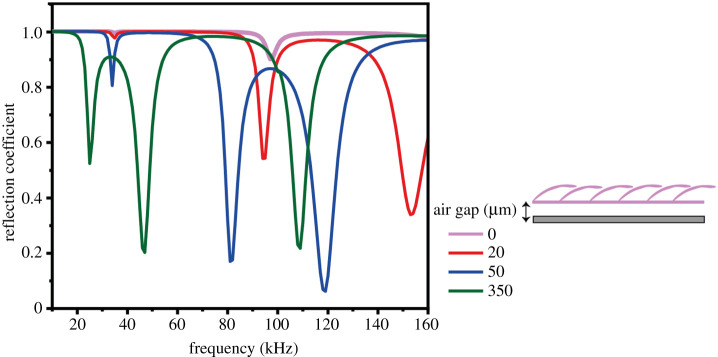
Effect of the depth of an air gap below a cover scale array on spectral RC for the **‘**Dorsal bald, ventral surface ensonified’ treatment. (Online version in colour.)

To understand our surprising finding that the orientation of a *Dorsal bald* sample on a metal disc drastically changed its acoustic absorption, we modelled the acoustic effect of an air gap underneath a *Dorsal bald* sample. We found that the modelled RC sinks are indeed sensitive to the presence and depth of an air gap (figure 5). An increase in the air gap (0, 20, 50 and 350 µm) apparently shifted the reflection sinks towards lower frequencies. The sinks also got deeper as a gap was introduced. Importantly, the same model repeated without scales showed an RC of 1 at all frequency without any sinks. This result corroborates the notion that the reflection sink on the RC spectra is due to the presence of scales.

### (c) Reflection directionality

#### (i) Measured directionality of target strength

**Figure 6.**
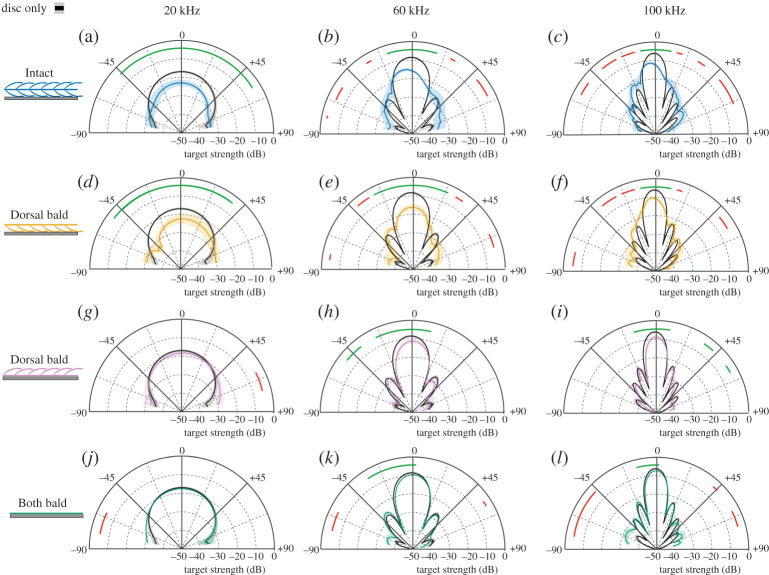
Directionality of target strength at 20, 60 and 100** **kHz (*n* = 5) between a metal disc (black) and a wing-covered disc, comparing (*a*–*c*) ‘Intact’, (*d*–*f*) ‘Dorsal bald, dorsal surface ensonified’, (*g*–*i*) ‘Dorsal bald, ventral surface ensonified’ and (*j–l*) **‘**Both bald’ wing samples. Shaded areas represent standard error. Coloured lines near edge of angle axis indicate significant pairwise differences (p ≤ 0.05; green lines = treatment is significantly less than metal disc, red line = metal disc is significantly less than treatment). (Online version in colour.)

At low ultrasonic frequencies (20 kHz), the reduction in target strength by an *Intact* sample ensonified on the dorsal surface is multidirectional, with a significant reduction in target strength at sound incident angles from −45° to +54° (figure 6*a*). As frequency increases, the effect on target strength becomes more directional, with significant target strength reductions covering −20° to +20° at 60 kHz and −8° to +11° at 100 kHz (figure 6*b,c*). Away from normal sound incidence and at higher frequencies, the wing-covered sample often showed a higher target strength than the bare metal disc. When the same *Intact* samples were ensonified on their ventral surface target strength was significantly reduced from −67° to +55° at 20 kHz, −11° to +22° at 60 kHz and −10° to +17° at 100 kHz (electronic supplementary material, figure S2). In the *Dorsal bald* dorsally ensonified treatment, the significant reduction in target strength covers the angles of −52° to +38° at 20 kHz, −25° to +22° at 60 kHz and −2° to +12° at 100 kHz (figure 6*d–f*). The same *Dorsal bald* samples ensonified ventrally, however, showed no reduction in target strength at 20 kHz at any angle (figure 6*g*), while there was a significant but small target strength reduction from −24° to +12° at 60 kHz and −19° to +12° at 100 kHz (figure 6*h,i*). The *Both bald* treatment showed no difference in target strength at 20 kHz (figure 6*j* and electronic supplementary material, figure S3), but a significant but biologically meaningless (less than 1 dB) reduction in target strength at −22° to +15° at 60 kHz and −18° to +15° at 100 kHz (figure 6*k,l*), with a nearly identical pattern when ensonified ventrally.

#### (ii) Modelled directionality of target strength

To investigate whether the observed directionalities could be a product of the scale layer resonances, we created a model matching the dimensions of the empirical measurements. Because the scales in the model are identical to each other, this model is only valid for resonance frequencies of this unit cell scale, which was found at 64 kHz. We therefore compare the model output with the measured directionality of the wing punch at that frequency.

**Figure 7.**
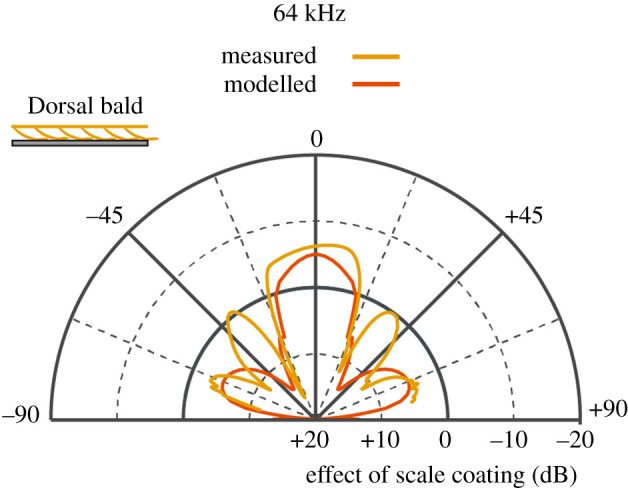
Directionality comparison showing the difference in target strength between the reflection from a metal disc and reflection from a scale array on a metal disk at 64** **kHz for both measured (gold) and modelled (orange) data in the dorsal bald, dorsal ensonified treatment. Note that negative dB means the moth wing reduces the reflections of the metal disc. (Online version in colour.)

The directionality in figure 7 shows the measured and modelled (at resonance frequency) acoustic effect of the scale layer on the reflections from the metal disc. Both measurement and model show the desired reduction in target strength for the main reflection lobe, but not for any side lobes. The modelled and measured width (±22.5°) and effect magnitude (−6 dB measured; −5 dB modelled) of the main reflection lobe are very similar. This supports the validity of our modelling approach and shows that the known resonant absorption mechanism can create the observed reflection directionalities.

## 4. Discussion

### (a) Acoustic performance

Our exploration of an evolved acoustic metamaterial as a potential concept for architectural acoustics revealed that an ultrathin (approx. 0.3 mm) coating by a natural moth wing significantly reduces the amount of acoustic energy reflected from a metal disk over a wide range of frequencies. The lowest RCs (0.13 to 0.27; figure 3) and biggest drop in RC were found at the longest wavelengths, giving the biological coating deep-subwavelength (1/50 of wavelength) performance, which is a desirable feature in architectural acoustics.

The acoustic effect is omnidirectional at low frequencies, with a significant reduction in target strength from −45° to +54° in the *Intact* sample at 20 kHz (figure 6). The effect becomes more directional towards higher frequencies, partly due to strong sidelobes in the ‘disc only’ treatment, which are less prevalent in a wing-covered disc. There is good agreement between the measured and modelled reduction in target strength at normal sound incidence (figure 7), being within 1 dB of each other. The poorer match outside the main lobe can likely be explained by the fact that measurements were from a circular sample covered in widely varying natural scales, while the model is a strip of the same diameter covered with only one type of scale. So differences in sidelobe pattern are to be expected.

Our measurement method only quantifies retroreflection as a measure of acoustic performance. Retroreflected acoustic energy could be reduced through increased transmission, absorption or/and diffusion. Transmission cannot increase in our experiment as the metal disc provides an acoustically hard, reflective surface. Thus, any changes in RC are brought about either by absorption or diffusion. Diffusion has previously been shown to be a non-significant factor in the acoustic performance of moth scales at normal sound incidence [19], meaning that the reduction in RC observed at 0° can be attributed to absorption. The observed minimum RC of 0.13 thus signifies a likely absorption coefficient of 0.87 (figure 3), which exceeds the maximum absorption coefficient of 0.72 documented previously [19]. This gain might relate to the fact that sound passes through the moth wing coating a second time after being reflected by the metal disc underneath. The present method for measuring oblique sound incidence angles (retroreflection) does not give a measure of diffusion, so it is unclear in what proportions the directionally broad reduction in reflection seen at lower frequencies (figure 6) can be attributed to increased absorption and/or diffusion. Both phenomena though (deep-subwavelength absorption and diffusion) are desirable material properties in architectural acoustics [23].

### (b) The influence of coating orientation

Moth wings as arrays of coupled resonant absorbers (individual scales) [19,21] can explain the acoustic performance of *Intact* samples observed here. Our unexpected finding that the orientation of a *Dorsal bald* sample affects its acoustic performance requires closer exploration. The key difference between dorsal and ventral ensonification is the freedom of movement of the wing membrane. When the dorsal surface was ensonified RC was reduced significantly (figure 3). In this case, the bald surface of the membrane faced the sound and was free to move itself, with the scales sitting between the membrane and the metal disc. In the opposite orientation, when the scales faced the sound and a thin layer of water created adhesion of the membrane to the metal disc, there was only a negligible effect on RC compared to the *Both bald* treatment (figure 3). We explored two possible explanations for this behaviour by FEM modelling.

The first potential explanation was that the membrane alone was the functional component and the scales simply created a gap between the membrane and the reflective surface underneath thereby constituting a conventional panel absorber of a resonating membrane working against air trapped underneath. The absence of a gap when ventrally ensonified would explain the lack of effect on the RC. When a bare wing membrane without scales was modelled with an air gap of different depths between the membrane and a reflective surface underneath (for gap depths figure 7) to test this hypothesis, there never was any absorption at any of the relevant frequencies tested here. This suggests that the wing membrane alone is not acting as a simple panel absorber [24,25] and that functionality requires scales attached to the membrane.

Our second potential explanation thus included scales as resonant elements. Confirming our measurements (figure 3), a ventrally ensonified *Dorsal bald* sample with an air gap below the membrane showed sinks in RC (figure 5) that changed in magnitude and sink frequency with gap depth. So the interaction between scale and the elastic membrane bearing them appears essential to dissipate acoustic energy. The resulting RC sinks were equivalent to those seen when the dorsal surface was ensonified (figure 4) and matched our RC measurements (figure 3). Without an air gap underneath the membrane, however, there was no effect on RC (figure 5) mirroring our measurements (figure 3). The exact mechanism by which the air gap affects RC is unclear, but likely includes a reduction in freedom of movement (effective elasticity) of the membrane by adhesion to the metal disc in combination with some gap depth-dependent tuning. We conclude that coupling between resonating scales and the elastic membrane brings about the correct conditions for energy dissipation. RC sinks of individual scales would create broadband absorption as a metamaterial array of tuned resonators following [19] with frequency tuning [26,27] by variation in scale morphologies creating their metamaterial functionality [28].

### (c) Similar metamaterials

The wing membrane and scale assembly of the moth wing bears some structural similarities to theoretical acoustic metamaterials. Membrane type acoustic metamaterials, consisting of decorated membrane resonators, have been shown to be very versatile in their application depending on certain design parameters, being capable of near-complete transmission [29], reflection [30–32] and absorption [33,34]. The mechanisms for these extraordinary material properties are a negative mass density [35] or negative refractive index [36,37], caused by subwavelength decorated membrane resonators. One such metamaterial consists of a platelet suspended on a membrane backed by a reflecting hard surface, between which a gas is sealed. The design allows the platelet and membrane resonator to oscillate, exhibiting two resonant modes, one of which relates to the platelet and one to the elastic membrane. The unique design of the decorated membrane absorber causes these two modes to hybridize, forming a new hybrid resonant mode where acoustic absorption is realized [4]. The design could be considered similar to the metal disc, moth wing membrane and scale system, with the moth wing membrane and scale able to oscillate about one another while suspended on the acoustically reflective metal disc.

### (d) Diffusion by the *Both bald* sample

There was a reduction in the RC towards higher frequencies in the *Both bald* treatment (minimum RC 0.64 at 160 kHz), and at the lowest frequencies the RC sometimes exceeded 1 (figure 3*c,d*; *Both bald*). The metal disc can be considered a smooth mirror reflector, to which the bald wing membrane on top adds a certain level of surface roughness. This roughness would diffuse some sound energy, which would explain the observed reduced RC at higher frequencies [38]. The fact that for shallow angles of incidence the RC increases (figure 6) further corroborates this interpretation.

## 5. Conclusion

Previous work has shown that air-backed moth wings demonstrate impressive sound absorption properties. Here we have shown that moth wings also function as sound absorbers when backed by an acoustically solid structure. The mechanism of sound absorption is unclear but is likely a combination of the mechanical absorption of the scales coupled with some dissipation through thermal and viscous effects brought about by the interaction of the scales, wing membrane and air movement through the scales. It is hoped that this understanding of the absorption mechanisms of scales of the moth wing will inspire the next generation of acoustic metamaterial sound absorbers.

## Data Availability

Data are available at the University of Bristol data repository, data.bris, at https://doi.org/10.5523/bris.ef1prjw40fhw2l20i9jmil5nq. The data are provided in the electronic supplementary material [39].
